# Therapeutic Potential of a Scorpion Venom-Derived Antimicrobial Peptide and Its Homologs Against Antibiotic-Resistant Gram-Positive Bacteria

**DOI:** 10.3389/fmicb.2018.01159

**Published:** 2018-05-29

**Authors:** Gaomin Liu, Fan Yang, Fangfang Li, Zhongjie Li, Yange Lang, Bingzheng Shen, Yingliang Wu, Wenxin Li, Patrick L. Harrison, Peter N. Strong, Yingqiu Xie, Keith Miller, Zhijian Cao

**Affiliations:** ^1^State Key Laboratory of Virology, College of Life Sciences, Wuhan University, Wuhan, China; ^2^Biomolecular Sciences Research Centre, Department of Biosciences and Chemistry, Sheffield Hallam University, Sheffield, United Kingdom; ^3^Department of Biology, School of Science and Technology, Nazarbayev University, Astana, Kazakhstan; ^4^Bio-drug Research Center, Wuhan University, Wuhan, China; ^5^Hubei Province Engineering and Technology Research Center for Fluorinated Pharmaceuticals, Wuhan University, Wuhan, China

**Keywords:** scorpion, venom peptides, antimicrobial peptides, activity and mechanism, antibiotic resistance

## Abstract

The alarming rise in the prevalence of antibiotic resistance among pathogenic bacteria poses a unique challenge for the development of effective therapeutic agents. Antimicrobial peptides (AMPs) have attracted a great deal of attention as a possible solution to the increasing problem of antibiotic-resistant bacteria. Marcin-18 was identified from the scorpion *Mesobuthus martensii* at both DNA and protein levels. The genomic sequence revealed that the marcin-18 coding gene contains a phase-I intron with a GT-AG splice junction located in the DNA region encoding the *N*-terminal part of signal peptide. The peptide marcin-18 was also isolated from scorpion venom. A protein sequence homology search revealed that marcin-18 shares extremely high sequence identity to the AMPs meucin-18 and megicin-18. *In vitro*, chemically synthetic marcin-18 and its homologs (meucin-18 and megicin-18) showed highly potent inhibitory activity against Gram-positive bacteria, including some clinical antibiotic-resistant strains. Importantly, in a mouse acute peritonitis model, these peptides significantly decreased the bacterial load in ascites and rescued nearly all mice heavily infected with clinical methicillin-resistant *Staphylococcus aureus* from lethal bacteremia. Peptides exerted antimicrobial activity via a bactericidal mechanism and killed bacteria through membrane disruption. Taken together, marcin-18 and its homologs have potential for development as therapeutic agents for treating antibiotic-resistant, Gram-positive bacterial infections.

## Introduction

Drug resistance among bacterial pathogens is becoming an increasingly serious problem for global public health and antimicrobial peptides (AMPs) hold promise as alternative treatment options to traditional antibiotics. AMPs are distributed among a wide range of species and represent an ancient host defense mechanism against infections in all living organisms, including bacteria, viruses, fungi, and parasites (Mygind et al., 2005; Dai et al., 2008; Zhao et al., 2009). AMPs are usually small, positively charged amphipathic molecules with diverse amino acid compositions and length (Gentilucci et al., 2006). Despite their vast structural diversity, most AMPs kill bacteria in similar ways, by membrane disruption or pore formation that induces an efflux of essential ions and nutrients (Melo et al., 2009). Their potency, low resistance rates and unusual mode of action make AMPs an attractive family of compounds with the potential to be developed as therapeutic agents.

Venomous animals (scorpions, snakes, spiders, etc.) are able to secrete toxins, both to capture prey and to protect themselves. Numerous scorpion venom peptides have previously been identified to have a range of antimicrobial activities (Harrison et al., 2014) including hadrurin (Torres-Larios et al., 2000), scorpine (Conde et al., 2000), pandinins (Corzo et al., 2001), IsCTs (Dai et al., 2001, 2002), opistoporins, parabutoporin (Moerman et al., 2002), mucroporin (Dai et al., 2008), imcroporin (Zhao et al., 2009), meucins (Gao et al., 2009, 2010), Im-1 (Miyashita et al., 2010), StCTs (Yuan et al., 2010; Cao et al., 2012b), ctriporin (Fan et al., 2011), HsAPs (Nie et al., 2012), VmCTs (Ramirez-Carreto et al., 2012), UyCTs (Luna-Ramirez et al., 2013), TsAPs (Guo et al., 2013), stigmurin (de Melo et al., 2015) and Smps (Harrison et al., 2016). These findings have indicated that AMPs from scorpion venoms can represent good candidates for the development of new antimicrobial drugs.

*Mesobuthus martensii* is probably the most well-characterized scorpion species in China. Three AMPs (BmKn2, BmKb1 and BmKbpp) have already been functionally characterized from the venom of *M. martensii* by our group. BmKn2 (13 amino acids, 1448 Da) has strong antimicrobial activity against both Gram-positive and Gram-negative bacteria, whereas BmKb1 (18 amino acids, 1910 Da) is only weakly inhibitory (Zeng et al., 2004). BmKbpp (47 amino acids, 5321 Da) was found to have better antimicrobial activity against Gram-negative bacteria than Gram-positive bacteria (Zeng et al., 2012).

Here we have characterized another AMP (marcin-18) from the venom of *M. martensii.* Based on the precursor nucleotide sequence of marcin-18 obtained from the venom, registered into GenBank (No. ADT89762.1) by Zhu’s lab, as well as the genome decoded by our own group (Cao et al., 2013), we have cloned and characterized the gene encoding marcin-18 by comparison of precursor nucleotide and genomic sequences. We have also used proteomic methods to identify the existence of marcin-18 in the venom and have evaluated the antimicrobial activity of marcin-18 and its homologs, both *in vitro* and *in vivo*. Their antimicrobial mechanism has also been investigated.

## Materials and Methods

### Genomic DNA Extraction and Gene Cloning

Scorpions (*M. martensii*) were purchased from Yimeng Mountain of Shangdong province. Genomic DNA was extracted from the legs of one scorpion using a TIANamp Genomic DNA Kit (Tiangen, Beijing) using proteinase K. Based on the *M. martensii* genome reported by our group, gene-specific primers were designed and synthesized (TIANYI HUIYUAN, Wuhan). To get the full-length genomic sequence of marcin-18, genomic DNA was amplified using the KOD-Plus-Neo Kit (TOYOBO, Japan) with the primers F/R (F: 5′-GGCGTACAACAGCTAATACGTT-3′; R: 5′-CACAACCGTTAGCTATTACCAG-3′). The PCR product was purified, inserted into the pClone007 Blunt Simple Vector (TsingKe, Beijing), and transformed into competent *Escherichia coli* DH5α cells. Positive clones were sequenced.

### Venom Collection

Crude venom was obtained by the electric stimulation of telsons (128 Hz, 20 V) and was collected into a 1.5 mL sterile Eppendorf tube. Physiological saline was used to enhance electrical contact. After lyophilization it was stored at -80°C.

### Separation of Venom Components by RP-HPLC

Crude venom components were pre-separated into two parts by 10 kDa Amicon Ultra-0.5 device (500 μL, Millipore, United States). The filtrate with molecular weight (MW) less than 10 kDa was loaded onto an analytical XDB-C18 reverse phase high performance liquid chromatography (RP-HPLC) column (Welch Materials, 5 μm, 4.6 × 150 mm). The column was eluted with 0.1% (v/v) trifluoroacetic acid (TFA) in Milli-Q water (solvent A) and 0.1% TFA in 90% acetonitrile (solvent B) at a flow rate of 1 mL/min using a linear gradient of 5–95% solvent B over 60 min, followed by 95% solvent B over 10 min. Elution was monitored by UV absorbance at 230 nm. Fractions were collected by appearing time of each peak. The eluate was pooled, lyophilized and stored at -20°C before used.

### LC/ESI-Q-TOF MS Analysis

The venom samples eluted from RP-HPLC with MW lower than 10 kDa were digested by trypsin and then loaded onto an Ultimate 3000 LC system (Dionex Ultrimate 3000), desalted and concentrated with 0.1% formic acid (FA) on a C18 PeoMap^TM^ precolumn. The peptide mixtures were separated by an analytical capillary C18 column with a linear gradient of 5–50% solvent C (99.9% acetonitrile with 0.1% FA) over 60 min at a flow rate of 300 nL/min. Peptides eluted from liquid chromatography (LC) were directly injected into the coupled quadrupole-time-of-flight mass spectrometry (Q-TOF MS) (microTOF-Q II, Bruker Daltonics, United States) with a nano-electrospray. The peptides were analyzed using the positive ion MS mode and data-dependent MS/MS mode used for survey scans to detect no less than three most abundant precursor ions. For collision-induced dissociation (CID) in MS/MS analysis, collision energy was selected automatically as a function of m/z and charge. The collision gas was argon. The temperature of the heated sample source was 150°C and the electrospray voltage was 1200 V. The MS/MS data acquired from LC/ESI-Q-TOF MS were matched against genomic and transcriptomic databases of *M. martensii* by Mascot searching and proteins were identified (Xu et al., 2014).

### Peptide Synthesis, Characterization and Sequence Analysis

The peptides used in this study were synthesized by GL Biochem (Shanghai, China) and amidated at the *C*-terminus with a purity of >95%. Peptide purity (>95%) was assessed by RP-HPLC with a semipreparative C18 column (Elite, 5 μm, 10 × 250 mm) using a linear gradient of 5–95% solvent B in 60 min at a constant flow rate of 5 mL/min. Their actual MWs were confirmed by matrix-assisted laser desorption ionization time-of-flight mass spectrometry (MALDI-TOF MS). A 1 μL aliquot of each peptide was spotted onto the target plate along with an equal volume of a matrix solution (10 mg/mL α-cyano-4-hydroxycinnamic acid (CHCA), 50% acetonitrile, and 0.1% TFA). The mixture was left to dry at room temperature. Mass spectrometry was performed by the FlexControl software (m/z range of 770–3500). The reflection mode and the accelerating voltage of 25 kV were chosen for work.

The helical wheel projection was performed online using the Heliquest analysis.^1^

### Circular Dichroism (CD)

CD measurements (ambient temperature) of each peptide at a final concentration of 100 μg/mL were recorded in water, 30% trifluoroethanol (TFE) and 70% TFE on a J-810 spectropolarimeter (Jasco, Tokyo, Japan) at 190–260 nm. Spectra were collected from three separate recordings.

### Bacterial Strains

*Staphylococcus aureus* AB94004, *Staphylococcus aureus* ATCC6538, *Staphylococcus aureus* ATCC25923, *Staphylococcus aureus* AB208193, *Staphylococcus epidermidis* AB208187, *Staphylococcus epidermidis* AB208188, *Micrococcus luteus* AB93113, *Bacillus thuringiensis* AB92037, *Bacillus subtilis* AB91021, *Escherichia coli* AB94012, *Pseudomonas aeruginosa* ATCC9027, and *Escherichia coli* ATCC25922 were purchased from the China Center of Type Culture Collection (CCTCC). Methicillin-resistant *Staphylococcus aureus* (MRSA) P1374, methicillin-resistant *Staphylococcus aureus* (MRSA) P1381, and penicillin-resistant *Staphylococcus aureus* (PRSA) P1389 were obtained from the 302nd military hospital of Beijing, China.

### Antimicrobial Assays

The antimicrobial activity was determined via a broth microdilution assay according to the procedure recommended by the Clinical and Laboratory Standards Institute with some modifications (Hou et al., 2011; Li et al., 2016). Bacteria were cultured in Luria-Bertani (LB) broth to OD_600_ = 0.6 at 37°C, then diluted to 10^5^–10^6^ CFU/mL in LB medium. Peptides were serially double diluted to a gradient concentration range from 48.3 to 1.5 μM in physiological saline. Eighty microliters of the bacterial suspension and 20 μL of diluted peptide at varying concentrations were added to 96-well plates, and then incubated for 16–18 h at 37°C with continuous shaking at 250 rpm. The antimicrobial activity of ampicillin and kanamycin were also tested as positive controls. Experiments were performed in triplicate. The minimum inhibitory concentration (MIC) was determined as the lowest peptide concentration at which no bacterial growth was observed.

### Salt and pH Stability Assays

Seventy micrograms of synthetic peptide was dissolved in 0, 50, 100, 150, 200, and 250 mM sterile NaCl solutions, respectively. MICs of the peptides against *S. aureus* AB94004 were determined with increasing salt concentrations. Experiments were performed in triplicate. In addition, the peptide in physiological saline was used as a control.

To determine pH stability, 70 μg peptide was adjusted to a pH range from 3.0 to 10.0 with the following sterile buffers according to previous protocol (Cao et al., 2015): 0.1 M citric acid-sodium citrate buffer (pH 3.0–5.0), 0.2 M sodium phosphate buffer (Na_2_HPO_4_-NaH_2_PO_4_) (pH 6.0–7.0), 0.05 M Tris–HCl buffer (pH 8.0–9.0), and 0.05 M glycine-NaOH buffer (pH 10.0). The antimicrobial activity of peptides against *S. aureus* AB94004 was tested using MIC assays.

### Mouse Peritonitis Model

Six to eight-week-old male BALB/c mice (18–21 g) were purchased from the Animal Center for Disease Control and Prevention (Hubei, China) and maintained under specific pathogen-free conditions at 25 ± 2°C and 50 ± 5% humidity, on a 12 h/12 h light/dark cycle.

All methods were performed in accordance with the relevant guidelines (Dai et al., 2008; Peng et al., 2017). The mice were intraperitoneally (i.p.) injected with 0.5 mL/mouse (5.85 × 10^7^ CFU) of the exponential phase MRSA P1381 inoculum in physiological saline containing 5% (wt/v) mucin. At 1 h after the bacterial infection, the mice were i.p. injected with 0.4 mL/mouse of physiological saline (I) (to serve as a negative control), marcin-18 peptide (10 mg/kg) (II), marcin-18 peptide (25 mg/kg) (III), meucin-18 peptide (25 mg/kg) (IV), megicin-18 peptide (25 mg/kg) (V), and vancomycin (25 mg/kg) (VI). The vancomycin dose was set based on previous studies (Cavanagh et al., 2013; Wang et al., 2018). Each group included 6 mice. After another 24 h post administration, subject peritonea were lavaged with 1 mL of physiological saline and lavage fluids were then collected. Ascites were diluted and plated on LB agar so that the number of bacterial colonies could be counted. The ability of the three peptides to rescue mice from lethal bacteremia was observed 2–4 times daily and survival was tracked for 7 days.

### Time-Killing Kinetics

*S. aureus* AB94004 were grown to exponential phase (OD_600_ = 0.5) and diluted to ca. 10^7^ CFU/mL in LB broth, prior to the addition of peptide solutions at 1 × MIC, 2 × MIC and 4 × MIC and kanamycin at 4 × MIC to the cell suspensions. Physiological saline and melittin were used as negative and positive controls. At each time point (0, 5, 15, 30 and 60 min), aliquots were taken and washed with physiological saline, and then diluted appropriately in the physiological saline. The surviving bacterial colonies were quantitated after the cells were spread on LB agar and incubated overnight at 37°C.

### Competition Assays

1 mg/mL of peptides were incubated with an equal volume of 5 mg/mL of the lipopolysaccharide (LPS, Sigma-Aldrich: L3012/L8643) or lipoteichoic acid (LTA, Sigma-Aldrich: L2515) for 30 min. The MICs of peptides treated with LPS or LTA against *S. aureus* AB94004 were subsequently measured.

### Fluorescence Measurements

*S. aureus* AB94004 were cultured to exponential phase (ca. 10^7^ CFU/mL), centrifuged and suspended in physiological saline. Then the fluorescence dye SYTOX green (Invitrogen) was added at a final concentration of 5 μM. After incubation for 10 min, 50 μL bacterial culture and equal volume of peptides serially diluted in physiological saline at final concentrations of 1 × MIC, 2 × MIC, and 4 × MIC were added in a Greiner 96-well flat-bottom black plate. Physiological saline and MSI 78 were used as negative and positive controls. Fluorescence was measured over 30 min at the excitation and emission wavelengths of 488 and 525 nm, respectively.

### Transmission Electron Microscopy (TEM)

The exponential phase bacteria *S. aureus* AB94004 (ca. 10^7^ CFU/mL) were incubated with peptides at the final concentration of 4 × MIC or without the peptides for 30 min at 37°C. Samples were harvested by centrifugation and washed with physiological saline. The samples were then semi-thin sectioned and examined by a Hitachi H-8100 transmission electron microscope.

### Statistical Analysis

The data were analyzed using the GraphPad Prism 6. Continuous variables are expressed as the mean ± standard error of the mean (SEM).

## Results

### Gene Organization and Structure of Marcin-18 From the Scorpion *M. martensii*

**FIGURE 1:**
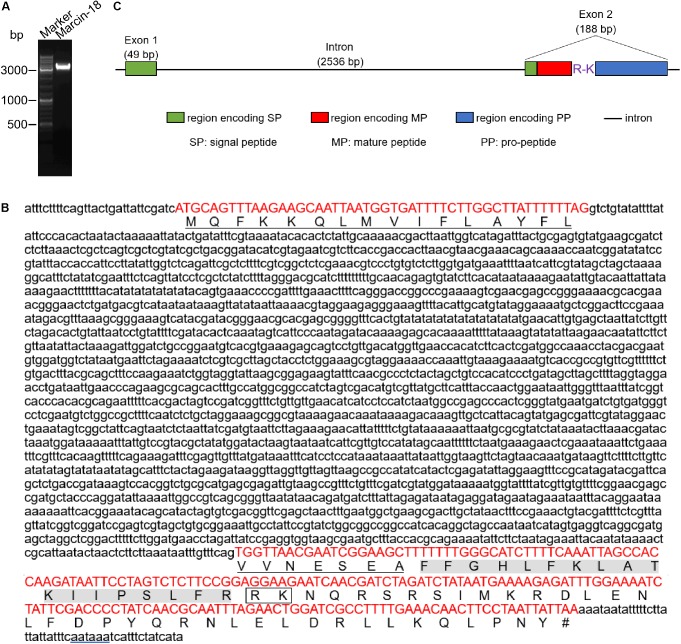
PCR amplification and genomic organization of marcin-18 coding gene. **(A)** PCR amplification of marcin-18 genomic region. Marker: 10 Kb DNA ladder. **(B)** Genomic sequence (above) and corresponding amino acid sequence (below) of marcin-18 gene. Red capital letters are signal peptide, mature peptide and propeptide coding regions. Lowercase letters are 5’ UTR, intron and 3’ UTR, respectively. The signal peptide residues are underlined. The mature peptide residues are shaded in gray. The potential post-translation cleavage site is marked in rectangle. The polyadenylation signal sequence (aataaa) is underlined in blue. Terminator is indicated by a pound sign (#). **(C)** The gene structure of marcin-18. The signal peptide (SP), mature peptide (MP) and pro-peptide (PP) are shown. Intron is designated by a straight line.

The precursor nucleotide sequence of marcin-18 has been registered into GenBank (No. ADT89762.1) by Zhu’s lab. The cDNA sequence consists of a 5′ UTR of 61 nt, an ORF of 237 nt, and a 3′ UTR of 68 nt. A single polyadenylation signal (aataaa) was found 13 nt upstream of the poly (A) tail. The ORF of 237 nt was predicted to encode a precursor that consists of 78 amino acid residues, containing a putative 23-residue signal peptide, a 37-residue propeptide and an 18-residue mature peptide (FFGHLFKLATKIIPSLFR) (**Figure 1B**). Multiple sequence alignments revealed that marcin-18 has high homology (94.44%) with meucin-18 (Gao et al., 2009) and megicin-18 (Diego-Garcia et al., 2014), AMPs from the venoms of the scorpions *Mesobuthus eupeus* and *Mesobuthus gibbosus*, respectively, suggesting that marcin-18 peptide may have antimicrobial activity.

To determine the gene organization and structure of marcin-18, we designed primers and amplified the genomic sequence of marcin-18 according to the genome of *M. martensii* decoded by our group (Cao et al., 2013). The PCR product was a single band with a size of approximately 3.5 kb (**Figure 1A**). The amplified DNA band was purified and sequenced. After DNA sequence analysis, the full-length genomic DNA sequence of the gene encoding marcin-18 was obtained (**Figure 1B**). A comparison of the genomic sequence with the corresponding cDNA sequence revealed that the gene contained a phase-I intron with a length of 2536 nt, located in the DNA region encoding the *N*-terminal part of signal peptide, which has a consensus GT-AG splice junction (**Figure 1C**). The A+T content of the intron is 62.78%, which is lower than that of both exon 1 (71.43%) and exon 2 (63.83%). The majority of toxin genes from scorpion venoms have a generic structure comprising two exons surrounding a single intron: the first exon encodes the first two-thirds of the signal peptide, whereas the second exon encodes the last third of the signal peptide, the mature peptide and the propeptide.

### Identification of Marcin-18 in the Venom of *M. martensii*

**FIGURE 2:**
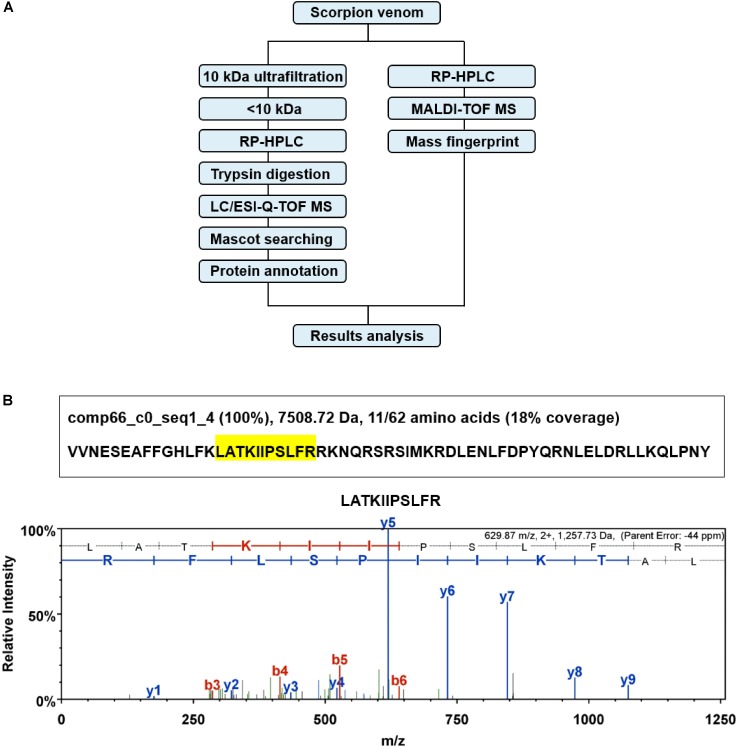
Identification of marcin-18 in the natural venom of the scorpion *M. martensii*. **(A)** Schematic overview of combined strategy for proteomic analysis of the *M. martensii* venom. **(B)** Identification of marcin-18 by ESI-Q-TOF MS combined with Mascot searching. The peptide sequence identified by Mascot searching was comp66_c0_seq1_4 from the transcriptome database of *M. martensii*. Protein identification probability, molecular weight and percentage of amino acids identified (shadowed in yellow) are shown. MS/MS spectrum of one amino acid sequence (m/z 629.87 (2+)) is shown. The amino acid sequence was confirmed by analyzing a_n_, b_n_, and y_n_ ions derived. The actual peptide mass is 1257.73 Da.

In order to identify the presence of marcin-18 in the crude venom of *M. martensii*, we employed a combined strategy of ultrafiltration and LC-MS (**Figure 2A**) to verify its existence. Firstly, the crude venom was separated into “high” and “low” MW fractions by ultrafiltration through a 10 kDa cut-off membrane. The “low” MW filtrate was then analyzed by RP-HPLC and LC/ESI-Q-TOF MS. All MS/MS data were collected and searched against genomic and transcriptomic databases of *M. martensii* by Mascot. Identified sequences were annotated by homology searching in the NCBI database combined with descriptions in UniProt. Marcin-18 (comp66_c0_seq1_4) was identified and displayed high-quality ESI-Q-TOF MS/MS spectrum (**Figure 2B**).

### Chemical Synthesis and Quality Analysis of Marcin-18 and Its Homologs

**FIGURE 3:**
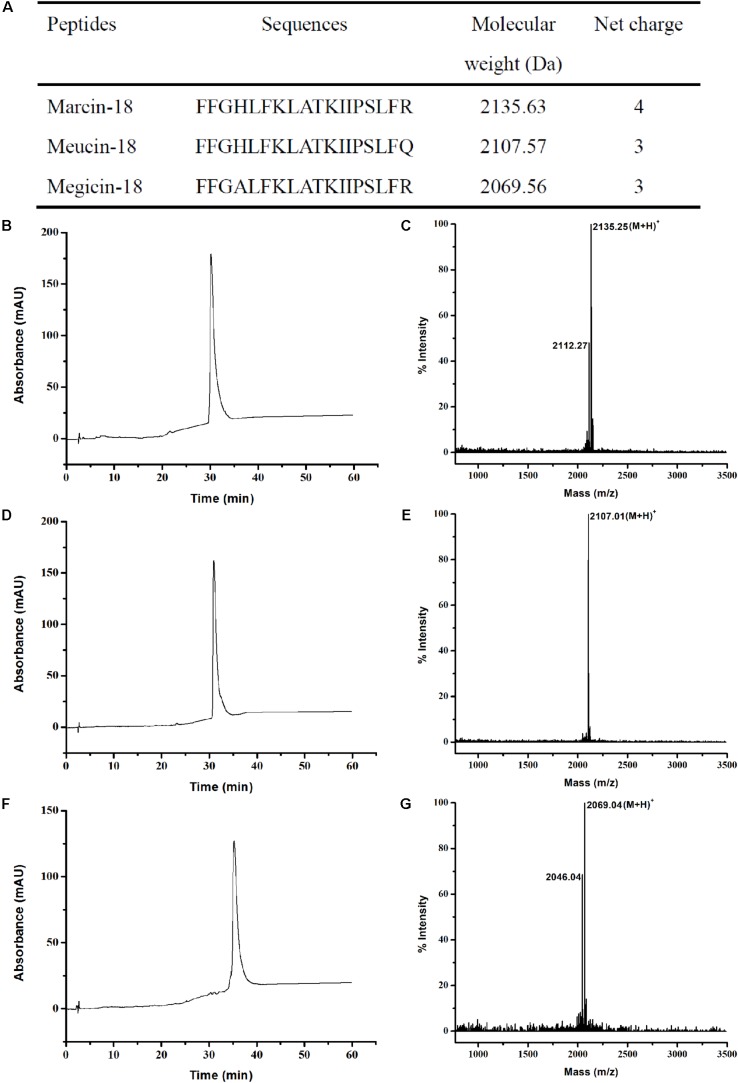
Chemical synthesis and characterization of marcin-18 and its homologs. **(A)** Physiochemical properties of marcin-18 and its homologs. RP-HPLC analysis of the synthetic marcin-18 **(B)**, meucin-18 **(D)** and megicin-18 **(F)**. Mass spectra of the synthetic marcin-18 **(C)**, meucin-18 **(E)** and megicin-18 **(G)** measured by MALDI-TOF.

Because of the extremely small amounts of marcin-18 in crude venom, the peptide was synthesized by solid state methods, in order to provide enough material for further studies. Bacterial recombinant expression systems are not very useful in this regard due to the inherent propensity of their products to lyse prokaryotic cell membranes. Marcin-18 and its homologs (meucin-18 and megicin-18) were synthesized using *N*-(9-fluorenyl) methoxycarbonyl (Fmoc) chemistry and *C*-terminal amidation. All peptides were >95% pure (HPLC) and verified by mass spectrometry: the calculated MW of marcin-18 was 2134.25, corresponding to its theoretical value 2134.63; the calculated MW of meucin-18 was 2106.01, corresponding to its theoretical value 2106.57; the calculated MW of megicin-18 was 2068.04, corresponding to its theoretical value 2068.56 (**Figure 3**).

### Structural Characteristics of Marcin-18 and Its Homologs

**FIGURE 4:**
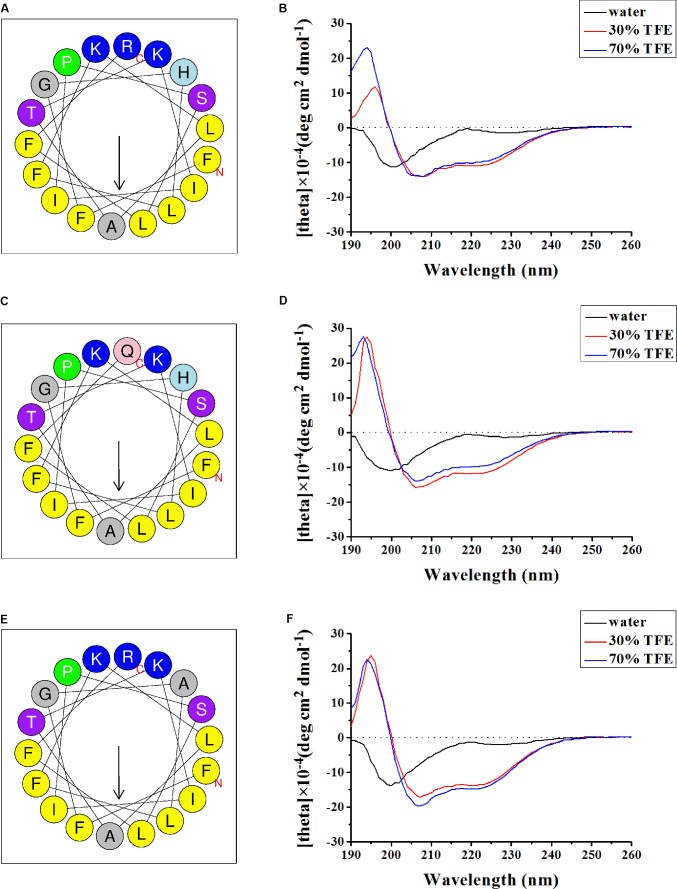
Secondary structure analysis of marcin-18 and its homologs. Helical wheel diagram of synthetic marcin-18 **(A)**, meucin-18 **(C)**, and megicin-18 **(E)** determined by the Heliquest method. The representation as a helical wheel shows the hydrophilic face and hydrophobic face. Basic (positive charged) residues are highlighted by blue background. Hydrophobic (non-polar) residues are highlighted by yellow background. Polar uncharged residues are highlighted by purple background. P (Pro) is a special residue shown by green background. CD spectra of marcin-18 **(B)**, meucin-18 **(D)**, and megicin-18 **(F)** (100 μg/mL) in water alone or with 30 or 70% aqueous TFE.

When plotted as helical wheel projections (**Figures 4A,C,E**) using the online program Heliquest (Gautier et al., 2008), marcin-18 and its homologs adopted an amphipathic structure, where hydrophobic residues are on one side while charged and neutral polar residues are found on the other side. On the hydrophilic side, Arg18 and His4 (marcin-18) are changed to Gln18 (meucin-18) and Ala4 (megicin-18), respectively. Structural differences between synthetic peptides, in either water or aqueous TFE, were confirmed by secondary structure analysis using CD spectroscopy (**Figures 4B,D,F**). All peptides exhibited random coil structures in water; however, they showed high percentages of α-helix in either 30 or 70% aqueous TFE. This suggests that peptides can form amphipathic α-helical structures in the appropriate membrane environment. Previous reports have suggested that hydrophobic and electrostatic interactions are the two main driving forces in peptide-membrane interactions (Shai, 1999; Yeaman and Yount, 2003). Our data suggests that marcin-18 and its homologs might follow this pattern.

### *In Vitro* Antimicrobial Activity of Marcin-18 and Its Homologs

The antimicrobial activities of marcin-18 and its homologs were studied and their MICs are shown in **Table 1**. With the exception of the Gram-positive *Micrococcus luteus* (MICs 23.4–48.3 μM), peptides generally had greater activities against Gram-positive organisms (MICs 1.5–3.0 μM) than against Gram-negative organisms (MICs 5.9–48.3 μM). Marcin-18 and its homologs were also tested against antibiotic-resistant bacteria. The antibiotic-resistant bacteria were clinical isolates, all of which were tested with traditional antibiotics to determine their resistance. MICs against PRSA and MRSA varied between 1.5 and 3.0 μM. These data suggest that these peptides are more effective in inhibiting antibiotic-resistant pathogens than many commercially available antibiotics (Cao et al., 2012a). MICs of AMPs (BmKn2, BmKb1 and BmKbpp) derived from *M. martensii* and antibiotics (ampicillin, kanamycin and vancomycin) are also shown (Zeng et al., 2004, 2012; Cao et al., 2012a). Marcin-18 and its homologs have greater antimicrobial activity than these other AMPs and some antibiotics.

**Table 1:** *In Vitro* antimicrobial activities of marcin-18 and its homologs compared with other peptides and antibiotics.

Strains	MIC (μM)								
	Marcin-18	Meucin-18	Megicin-18	BmKn2	BmKb1	BmKbpp	Amp	Kana	Van
**Gram-positive bacteria**								
*Staphylococcus aureus* AB94004	1.5	1.5	1.5	4.3	–	–	67.3	5.4	–
*Staphylococcus aureus* ATCC6538	1.5	1.5	1.5	–	–	–	–	–	–
*Staphylococcus aureus* ATCC25923	2.9	3.0	3.0	4.3	–	–	–	10.7	–
*Staphylococcus aureus* AB208193	2.9	3.0	3.0	–	–	–	–	–	–
*Staphylococcus aureus* ATCC14458	–	–	–	–	–	> 70	–	–	–
*Staphylococcus aureus*	–	–	–	–	8.4	–	–	–	–
*Staphylococcus epidermidis* AB208187	2.9	3.0	1.5	–	–	–	–	–	–
*Staphylococcus epidermidis* AB208188	2.9	1.5	1.5	–	–	–	–	–	–
*Micrococcus luteus* AB93113	23.4	47.5	>48.3	4.3	42.6	–	–	–	–
*Micrococcus luteus* ATCC9341	–	–	–	–	–	20.3	–	–	–
*Bacillus thuringiensis* AB92037	2.9	3.0	1.5	8.6	–	–	–	–	–
*Bacillus subtilis* AB91021	2.9	3.0	3.0	8.6	25.5	–	–	42.9	–
*Bacillus subtilis* ATCC6051	–	–	–	–	–	24.4	–	–	–
*Listeria monocytogenes* ATCC35152	–	–	–	–	–	5.7	–	–	–
*Enterococcus faecalis* ATCC14508	–	–	–	–	–	57.1	–	–	–
*Nocardia asteroids* ATCC3308	–	–	–	–	–	>70	–	–	–
**Gram-negative bacteria**								
*Escherichia coli* AB94012	11.7	23.7	>48.3	>69	–	–	–	–	–
*Escherichia coli* ATCC25922	5.9	11.9	12.1	>69	–	–	33.7	21.5	–
*Escherichia coli* DH5α	–	–	–	–	–	2.3	–	–	–
*Escherichia coli*	–	–	–	–	9.5	–	–	–	–
*Pseudomonas aeruginosa* ATCC9027	5.9	11.9	12.1	–	–	–	–	–	–
*Pseudomonas aeruginosa* AB93066	–	–	–	34.5	–	–	–	–	–
*Pseudomonas aeruginosa* ATCC9229	–	–	–	–	–	4.7	–	–	–
*Pseudomonas aeruginosa*	–	–	–	–	47.5	–	–	–	–
*Haemophilus influenzae* ATCC31517	–	–	–	–	–	3.2	–	–	–
*Klebsiella pneumoniae* ATCC13882	–	–	–	–	–	2.8	–	–	–
*Salmonella enterica* ATCC8090	–	–	–	–	–	2.3	–	–	–
*Serratia marcescens* ATCC13880	–	–	–	–	–	68.2	–	–	–
**Penicillin resistant bacteria**								
PRSA P1383	–	–	–	8.6	–	–	–	–	4.2
PRSA P1389	2.9	3.0	1.5	8.6	–	–	–	–	4.2
**Methicillin resistant bacteria**								
MRSA P1369	–	–	–	8.6	–	–	–	–	4.2
MRSA P1374	2.9	3.0	3.0	8.6	–	–	–	–	4.2
MRSA P1381	1.5	1.5	3.0	8.6	–	–	–	–	2.1
MRSA P1386	–	–	–	8.6	–	–	–	–	2.1

### Effect of Salt and pH on Activity of Marcin-18 and Its Homologs

**FIGURE 5:**
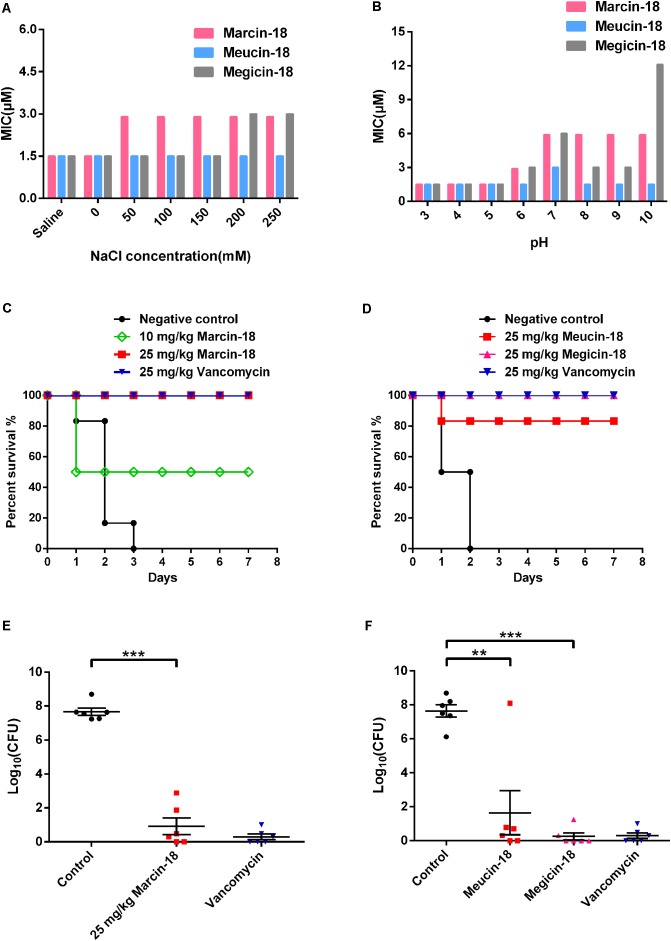
*In vitro* salt/pH stability and *in vivo* antimicrobial activity of marcin-18 and its homologs. **(A,B)** Effect of salt and pH on the antibacterial activity of marcin-18 and its homologs. **(C,D)** Therapeutic efficacy of marcin-18 and its homologs on MRSA infected mice. Vancomycin was used as the antibiotic control. There were six mice in each group. The infected mice were inspected 2–4 times each day and survival were tracked for 7 days. **(E,F)** Quantitative comparison of peritoneal bacterial counts of mice treated with physiological saline versus those of mice treated with peptides at 24 h after peptide administration. Physiological saline was used as the negative control. Each point represents data from a single mouse. Mean values are presented, *n* = 6. Error bars indicate ± SEM. The data were subjected to a one-way analysis of variance (ANOVA) and significant differences between the means were evaluated. A probability value of < 0.05 was considered significant. ^∗∗^*p* < 0.01 for physiological saline versus meucin-18. ^∗∗∗^*p* < 0.001 for physiological saline versus marcin-18 and megicin-18.

The three peptides retained stable antibacterial activity against *S. aureus* AB94004 in the NaCl range of 0–250 mM (**Figure 5A**). Meanwhile, marcin-18 and megicin-18 exhibited stable activity against *S. aureus* AB94004 in an acidic environment (pH 3.0–5.0), but it was reduced in neutral and alkaline environments (pH 6.0–10.0). Meucin-18 has great tolerance to the acidic, neutral and alkaline environments (**Figure 5B**).

### *In Vivo* Antimicrobial Activity of Marcin-18 and Its Homologs

We further explored the antibacterial and protective ability of marcin-18 and its homologs in a mouse model of lethal peritonitis. The optimally infective dose for BALB/c mice was determined to be approx. 10^8^ CFU/mL. Mice were inoculated intraperitoneally with 5.85 × 10^7^ CFU of MRSA P1381, followed by intraperitoneal treatment with a single dose of synthetic peptide or vancomycin (*n* = 6) at 1 h after bacterial injection. The mice were inspected for 7 days. As shown in **Figure 5C**, all six mice in the negative control group (MRSA, no peptide, physiological saline) died after infection within 3 days, but half the mice treated with marcin-18 (10 mg/kg) survived the study period. 100% of the mice survived in groups that had been treated, either with marcin-18 (25 mg/kg) or with vancomycin (25 mg/kg). In a separate experiment with marcin-18 analogs, 83.3% (5/6 mice) survived after treatment with meucin-18 (25 mg/kg) and 100% of mice survived after treatment with megicin-18 (25 mg/kg) (**Figure 5D**). In all cases, the surviving mice were unaffected and behaved normally.

The success of this treatment was also evaluated by the decrease in peritoneal bacterial counts of peptide-treated mice compared with untreated control mice and vancomycin-treated mice. 24 h after the bacterial infection, the bacterial load in the ascites of control mice was approximately 10^8^ CFU. Administration of marcin-18 significantly reduced the bacterial load in ascites by about 5 × 10^6^ CFU compared with that of mice in the physiological saline-treated group (*P* < 0.001) (**Figure 5E**). The treatments with meucin-18 and megicin-18 markedly decreased the bacterial load of mice with bacteremia by approx. 10^6^ CFU (*P* < 0.01) and 10^7^ CFU (*P* < 0.001) respectively (**Figure 5F**), compared with those of control mice. Mice in the antibiotic-treated group had few detectable bacteria. Collectively, these results suggest that marcin-18 and its homologs have antimicrobial activity *in vivo*, as well as suggesting that the peptides, over the time period studied, are not cytotoxic.

### Antibacterial Mechanism of Marcin-18 and Its Homologs

**FIGURE 6:**
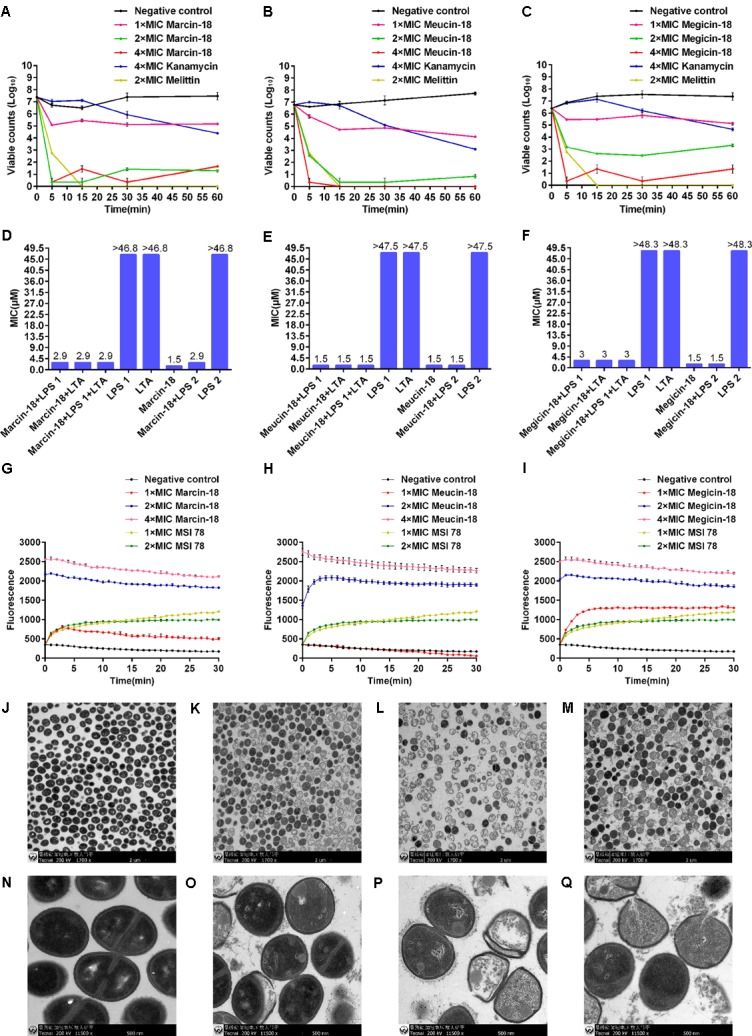
Antibacterial mechanism of marcin-18 and its homologs. Time-killing kinetics of marcin-18 **(A)**, meucin-18 **(B)** and megicin-18 **(C)** against *S. aureus* AB94004. The assay was performed by determining the counts of surviving bacteria. 0 h represents bacteria before treatment. Negative controls: physiological saline and kanamycin. Melittin was used as a positive control. The MICs of kanamycin and melittin against *S. aureus* AB94004 were 5.4 μM and 2.2 μM. MICs of marcin-18 **(D)**, meucin-18 **(E)** and megicin-18 **(F)** treated with LPS or LTA against *S. aureus* AB94004. LPS 1 and LPS 2 are from *E. coli* and *P. aeruginosa*. Fluorescence measurements of marcin-18 **(G)**, meucin-18 **(H)** and megicin-18 **(I)**. Negative control: physiological saline. MSI 78 is an amphipathic α-helical peptide with an antibacterial mechanism of disrupting the membrane. The MIC of MSI 78 against *S. aureus* AB94004 was 2.5 μM. Mean values are presented, *n* = 3. Error bars indicate ± SEM. Transmission electron microscopy analysis of *S. aureus* AB94004 treated with marcin-18 **(K,O)**, meucin-18 **(L,P)** and megicin-18 **(M,Q)**. **(J,N)** Negative control. Treatment with the peptides at the concentration of 4 × MIC for 30 min.

To gain insights into the antibacterial mechanism of marcin-18, meucin-18, and megicin-18, *S. aureus* AB94004 was used as a model bacterium to investigate the exact mode of action of these peptides. Using a time-killing assay, the killing rate increased with increasing peptide concentration. Marcin-18 (**Figure 6A**) and meucin-18 (**Figure 6B**) rapidly killed bacterial cells and achieved an approx. 2-lg reduction in 5 min and within 15 min at 1 × MIC. Viable colony number decreased approximately 15% in 5 min at 1 × MIC in the presence of megicin-18 (**Figure 6C**).

To determine whether LPS or LTA interfered with the antibacterial activities of marcin-18, meucin-18, and megicin-18, peptides were mixed with LPS or LTA and MIC values against *S. aureus* were re-measured. MICs of marcin-18 (**Figure 6D**) and megicin-18 (**Figure 6F**) were reduced by only two-fold and meucin-18 (**Figure 6E**) was unchanged. These results suggest that the LPS from *E. coli* (LPS 1) or *P. aeruginosa* (LPS 2) and LTA from *S. aureus* do not significantly affect the antimicrobial properties of the three peptides.

To further assess whether these peptides could damage the integrity of the bacterial cell membrane, *S. aureus* were pre-treated with the fluorescent nucleic acid stain SYTOX green before incubating with each of the peptides in separate experiments. The fluorescence had a rapid increase over 5 min at 1 × MIC of marcin-18 (**Figure 6G**) and megicin-18 (**Figure 6I**) and 2 × MIC of meucin-18 (**Figure 6H**) when *S. aureus* cells were exposed to SYTOX and the peptides, compared with a stable increase upon exposure to MSI 78 (the positive control). However, the fluorescence did not have an apparent increase over 30 min at 1 × MIC of meucin-18 (**Figure 6H**). When treated with 2 × MIC and 4 × MIC of marcin-18 (**Figure 6G**) and megicin-18 (**Figure 6I**), the fluorescence reached to the maximum value before detected. These results indicated that these peptides killed the bacteria by fastly disrupting the bacterial membrane.

To determine the direct influence of these peptides on the bacteria, TEM was used to examine the ultrastructural changes of *S. aureus* after treatment with peptides. Untreated cells of *S. aureus* were round, proliferating cell with intact cell wall and well-defined membrane (**Figures 6J,N**). After treatment (4 × MIC) with either marcin-18 (**Figures 6K,O**), meucin-18 (**Figures 6L,P**), or megicin-18 (**Figures 6M,Q**) for 30 min, disruption of the cell envelope was observed, together with the appearance of a granulated cytoplasm. These morphological changes support our previous experiments, providing evidence for peptide-induced disruption of the bacterial plasma membrane.

## Discussion

There is an urgent need for more effective agents to overcome drug resistance in bacteria. In addition to the three AMPs (BmKn2, BmKb1, and BmKbpp) already characterized from the venom of the Chinese scorpion *M. martensii*, we have identified and functionally characterized a new AMP (marcin-18). Using the marcin-18 precursor sequence as a query to blast in the NCBI database, we found high similarity to precursors of meucin-18 and megicin-18, AMPs from *M. eupeus* and *M. gibbosus*, respectively. In the present study, we have synthesized the three peptides and investigated their antimicrobial activity and mechanism action.

Marcin-18 and its homologs had a wide spectrum of antimicrobial activity *in vitro* against Gram-positive and Gram-negative bacteria. They had better inhibitory activity against Gram-positive bacteria than Gram-negative bacteria. It is tempting to suggest that differences in the molecular composition of Gram-positive vs Gram-negative bacterial membranes are responsible for this selectivity although at present we cannot offer any meaningful insights in this regard. Our *in vivo* study with a mouse model of lethal peritonitis has also demonstrated that peptide-treated mice have a high survival rate, suggesting that these peptides were still active in the peritoneal cavity.

Our data suggests that major negatively charged components of the prokaryotic cell wall (e.g LPS and LTA) do not have a detrimental effect on the antimicrobial activity of marcin-18 and its homologs. In contrast to some previous results (Fjell et al., 2011) our studies also indicate that LPS and LTA are not the primary site of antimicrobial action of these peptides.

Structure analysis suggested that marcin-18 and its homologs are amphipathic, cationic, α-helical peptides. Usually, natural AMPs with such structures are considered to be membrane-lytic peptides (Yamaguchi et al., 2001; Yang et al., 2001), which kill bacteria within 2–3 min of initial exposure (Hartmann et al., 2010; Huang et al., 2010). They bind to bacterial membranes and often non-specifically increase membrane permeability, resulting in the leakage of intracellular components (Peng et al., 2017). The rapid degradation of *S. aureus*, as evidenced by cytoplasmic membrane permeation assays and TEM have demonstrated that marcin-18 and its homologs are classical membrane-lytic peptides. Though these cationic peptides do not interact with either LPS or LTA, there are many other negatively charged molecules on the outer surface of the bacterial cell; electrostatic interactions between the peptides and the bacterial surface will encourage these peptides to pass through the cell envelope (Brogden, 2005).

Although AMPs such as marcin-18 and its homologs have potential advantages over conventional antibiotics, since their mechanism of action makes the development of bacterial resistance more unlikely, obstacles still remain in their progression to drugs of therapeutic usefulness. One of the biggest challenges is to develop AMPs that selectively permeabilize bacterial membranes and therefore eliminate potential cytotoxic effects in mammalian cells. Our studies with the lethal peritonitis mouse model, demonstrating that marcin-18 and its homologs can rescue mice from a lethal dose of MRSA, offers great promise in this regard.

## Ethics Statement

All animal experiments were carried out in accordance with the recommendations and approved by the Institutional Animal Care and Use Committee of Wuhan University.

## Author Contributions

All authors listed have made a substantial, direct and intellectual contribution to the work, and approved it for publication.

## Conflict of Interest Statement

The authors declare that the research was conducted in the absence of any commercial or financial relationships that could be construed as a potential conflict of interest. The reviewer MR and the handling Editor declared their shared affiliation.

## Footnotes

1 http://heliquest.ipmc.cnrs.fr/cgi-bin/ComputParamsV2.py
